# *LRR1* involved in the abscisic acid signaling pathway to regulate the early growth and development of *Arabidopsis thaliana*

**DOI:** 10.7717/peerj.18460

**Published:** 2024-11-26

**Authors:** Xiaoyang Xie, Lei Wei, Hongyuan Han, Bingnian Jing, Yuqing Liu, Yong Zhou, Ningjie Li, Xiao Li, Wei Wang

**Affiliations:** 1 Key Laboratory of Natural Products, Henan Academy of Sciences, Zhengzhou, Henan Province, China; 2 State Key Laboratory of Cotton Biology, School of Life Sciences, Henan University, Kaifeng, Henan Province, China; 3 Henan Natural Products Biotechnology Co., Ltd, Zhengzhou, Henan Province, China; 4 Center of Quality Inspection and Analysis Assaying Research, Henan Academy of Sciences, Zhengzhou, Henan Province, China

**Keywords:** Abiotic stresses, LRR-RLKs, Abscisic acid signling, *Arabidopsis thaliana*, Early growth

## Abstract

**Background:**

Living organisms possess the remarkable capacity to swiftly adapt to fluctuations in their environment. In the context of cell signal transduction, a significant challenge lies in ensuring the effective perception of external signals and the execution of appropriate responses. To investigate this phenomenon, a recent study utilized *Arabidopsis thaliana* as a model plant and induced stress by administering abscisic acid (ABA), a plant hormone, to elucidate the involvement of leucine-rich repeat receptor-like kinase1 (LRR1) in ABA signaling pathways.

**Methods:**

Homozygous T-DNA insertion alleles for *LRR1* and *KIN7* were isolated. Quantitative real-time PCR (qRT-PCR) was performed to confirm the expression of the *LRR1* gene. Subcellular localization and beta-glucuronidase (GUS) tissue labeling techniques were utilized to determine the expression pattern of the *LRR1* gene in cells and tissues. Yeast two-hybrid complementation, bimolecular fluorescence complementation assay, and GST pull-down assays were conducted to validate the interaction of LRR1 proteins.

**Results:**

Phenotypic analyses revealed that *lrr1* and *kin7* mutants are less sensitive to the inhibitory effects of ABA on germination and cotyledon greening that is seen in WT. Mutants *LRR1* and kinase 7 (KIN7) exhibited resistance to ABA and displayed normal growth patterns under control conditions. The double mutant *lrr1kin7* showed reduced responsiveness to ABA. Conversely, overexpression lines *LRR1ox2* and *LRR1ox10* demonstrated heightened sensitivity to ABA, resulting in severe growth reduction. qRT-PCR assay indicated that exogenous application of ABA led to significant down-regulation of *ABI3*, *ABI4*, and *ABI5* transcription factors in *LRR1* material compared to wild-type WT material. An investigation was conducted to determine the expression pattern and transcriptional level of *LRR1* in Arabidopsis. The results revealed ubiquitous expression of *LRR1* across all developmental stages and tissue tested. Subcellular localization assays confirmed the presence of LRR1 on the plasma membrane of cells. Furthermore, BiFC assay, yeast two-hybrid complementation, and GST pull-down assays demonstrated an interaction between LRR1 and PYL6 *in vitro*. These findings provide substantial insights into the involvement of *LRR1* in the ABA signaling pathway while regulating seed germination and cotyledon greening during early development in Arabidopsis. This study significantly advances our understanding regarding the correlation between *LRR1* and ABA signaling pathways with potential applications for enhancing crop stress resistance.

## Introduction

As sessile organisms, plants exhibit distinct characteristics from animals as they confront diverse environmental challenges encompassing biotic and abiotic stresses. Among these, abiotic stresses such as drought and high salinity profoundly impact plant growth, development, and global crop production. To cope with these challenges, plants have evolved multiple adaptive strategies involving the transduction of stress signals to activate stress-responsive genes while modulating metabolic rate and ion channel permeability (Emenecker & Strader, 2020; Pan et al., 2020; Shimotohno et al., 2021). Among these strategies, ABA plays a significant role by delaying seed germination, cotyledon greening, and plant growth, reducing transpiration rate, and regulating cellular water potential (Wang et al., 2020). It is one of the essential hormones in plants (Sano & Marion-Poll, 2021; Pan et al., 2021; Chen et al., 2020). As a “stress hormone”, ABA also plays a crucial role in coping with various environmental stresses during plant seed germination. Under water stress, the content of ABA in plant cells increases rapidly, which promotes stomatal closure and regulates the expression of a large number of related genes, thus reducing the damage to plant tissues caused by drought or high salt stress (Yoshida et al., 2019a). At the same time, some ABA-deficient and ABA-insensitive mutants have obvious defective phenotypes in development, and their seed proliferation is seriously impaired even under adequate water conditions (Nakashima et al., 2009).

As a pivotal regulatory factor, the plant hormone ABA plays a crucial role in governing various aspects of plant growth, development, and physiological processes encompassing seed germination and dormancy, seedling growth and development, as well as numerous abiotic stress responses (Zhu et al., 2020; Zhang et al., 2020; Nagar et al., 2022). ABA-responsive mutants were previously obtained by genetic screening in seed germination assays and confirmed to be less sensitive to exogenous ABA, such as the dominant alleles ABA-INSENSITIVE 1 (*abi1-1*) and *ABI2 (abi2-1)* and the recessive alleles of *abi3*, *abi4*, *abi5* in *Arabidopsis thaliana (L.) Heynh* (Peng et al., 2022; Li et al., 2020; Collin, Daszkowska-Golec & Szarejko, 2021). While *abi1-1* and *abi2-1* impair many ABA signaling responses, including inhibiting seed germination, seedling growth, and promoting stomatal closure, *abi3*, *abi4*, and *abi5* demonstrate their reduced ABA sensitivity during seed germination and seedling growth (Utsugi et al., 2020; Jia et al., 2021). As diversion factors, *ABI3*, *ABI4*, *and ABI5* are mainly expressed in seeds, but their expression levels are relatively low in plant tissues (Liu et al., 2021). ABI1 and ABI2, as protein phosphatases (PP2Cs), play a negative regulatory role in the ABA signaling pathway (Wang et al., 2018; Leung, Merlot & Giraudat, 1997). It has also been reported that a protein kinase family SnRKs in the ABA signaling pathway has positive regulatory functions and regulates stomatal response ABA signaling (Nakashima et al., 2009; Ali et al., 2019).

ABA receptors play a crucial role in the ABA signaling pathway, facilitating recognition of ABA signals and initiating the primary process of signal transduction. In 2009, two independent groups identified ABA receptors, PYR/PYL/RCAR proteins, in Arabidopsis using mutant screening and yeast two-hybrid methods, respectively (Park et al., 2009; Ma et al., 2009). The central role of PYL/PYR/RCAR proteins in ABA perception was subsequently confirmed by the crystal structure of the PYR1-ABA complex (Santiago et al., 2009). Many reviews have been written on ABA signaling pathways (Fujita et al., 2011; Daszkowska-Golec & Szarejko, 2013). In the presence of ABA, PYR/PYL/RCAR proteins lead to inhibition of PP2C activity and disinhibition of SnRK2 protein kinase to activate ABA-responsive binding factors (ABFs)/ABA-Responsive Element Binding Proteins (AREBs) (Zhao et al., 2018a; Zhao et al., 2018b). The subsequent ABA response is mediated by many transcription factors, suggesting that the transcriptional cascade is the basic framework for ABA signal transduction in the nucleus; in the cytoplasm of guard cells, ABA activates outward ion channels, which results in stomata closure (Zhao et al., 2020).

Living organisms’ ability to respond quickly to external stimuli is essential. One problem to be solved in cell signal transduction is how the organism senses the external signal and produces the response (Zhu et al., 2023). In plants, many proteins are similar in structure and function to animal receptor protein kinases. According to reports, structural analysis shows that these proteins are mostly serine/threonine kinases, but their functions are still uncertain. They are called receptor-like protein kinases (RLKs) (Becraft, 2002). The largest subfamily of the RLKs family is the leucine-rich repeat (LRR-RLKs) subfamily, which includes more than half of the members of receptor-like protein kinases (Man, Gallagher & Bartlett, 2020; Liu et al., 2017; Fischer et al., 2016; Dufayard et al., 2017). LRR-RLKs belong to the transmembrane class of receptor protein kinases, which bind to ligands *via* extracellular receptor domains and amplify signaling cascades *via* intracellular kinase domains (Fan et al., 2018; He et al., 2018; Jamieson, Shan & He, 2018).

Phytohormones regulate plant growth and development. The hormone can bind to the receptor as a ligand, thus activating the signal transduction process (Smakowska-Luzan et al., 2018). RPK1 is one LRR-RLK in Arabidopsis. Localized in the cell membrane, its expression is up-regulated by ABA, dehydration, hyper-salinity, and low temperature (Osakabe et al., 2010; Rahim et al., 2022). The absence of *RPK1* resulted in the insensitivity of plants to ABA concerning seed germination, plant growth, stomatal closure, and gene expression (Shang et al., 2020). These findings suggest that *RPK1* may be a positive regulator of the ABA signaling pathway, regulating various ABA responses in growth, development, and stress response (Lee et al., 2011). Studies have shown that the expression levels of most ABA-induced genes, such as *ADHI*, *RD22*, *ERD4*, *etc.*, are significantly reduced in *rpk1* mutants. Therefore, *RPK1* is involved in regulating ABA-induced gene expression as part of the ABA signaling pathway (Fiesselmann et al., 2015). The signal transduction of brassinosteroid (BR) requires the interaction between BRI1 and BAK1 to form the receptor complex. BRI1 is an RLKs with 25 LRR. BRI1 senses the BR signal through its extracellular domain and conducts downstream signal transduction through kinase activity. The foreign signal is transmitted to the cell through the receptor protein kinase, which causes a series of cell responses (Lozano-Elena & Caño Delgado, 2019; Jaillais & Vert, 2016; Samakovli et al., 2022; Li, Zhang & Wang, 2017).

At present, the studies on the mode of action and downstream transcription factors of receptor genes *ERATA*, *CLV1*, and *BRI1* in the process of signal transduction are recently intensive (Hohmann et al., 2018; Je et al., 2018; Guo et al., 2018; Nguyen et al., 2019). A recent report showed that the loss-of-function mutations in two Arabidopsis receptor-like protein kinases, LRR1 and KIN7, were more sensitive to drought stress than the wild-type (Chen et al., 2021).

In this study, we isolated and screened T-DNA insertion mutants of *LRR1* and obtained transgenic plants overexpressing *LRR1* through genetic transformation. Physiological and cell biological investigations of *LRR1*, a member of the leucine-rich repeat receptor-like kinase family in Arabidopsis, have revealed its involvement in early growth and development processes, particularly in positively regulating seed germination and cotyledon greening within the context of ABA signaling. We collected Arabidopsis genes with similar sequences to *LRR1* from the TAIR website (https://www.arabidopsis.org) and constructed an evolutionary tree, which identified a highly homologous protein named *KIN7* that may potentially function redundantly with *LRR1*. Consequently, we created double mutant *lrr1kin7* to perform biochemical and molecular analyses that further confirmed the identity of *LRR1* while highlighting its role in the ABA signaling pathway. This study holds significant theoretical implications for elucidating the relationship between *LRR1* and ABA signaling pathways as well as practical value for enhancing crop stress tolerance.

## Materials and Methods

### Plant materials and growth conditions

The Columbia-0 ecotype of Arabidopsis was used in all assays. The *LRR1* (At5g16590) T-DNA insertion line SALK_053366 (*lrr1-1*), SAIL_412_D10 (*lrr1-2*), and the *KIN7* (At3g02880) T-DNA insertion line SALK_001905 (*kin7*) were obtained from the European Arabidopsis Inventory Center. The genotyping primers were used as described in Table S1. Seeds were germinated at 22 °C in a standard growth chamber with a 14 h/10 h light/dark cycle. 10-day-old seedlings grown on 1/2 MS plates were transplanted into the soil in a phytochamber with a 16-h/8-h light/dark cycle at 22 °C. we isolated and screened *lrr1-1*, *lrr1-2*, *kin7* mutant plants, and constructed *LRR1ox2*, *LRR1ox10* overexpression lines, construction of the double mutant *lrr1kin7* by single mutants *lrr1-1* and *kin7* of *LRR1* and *KIN7*.

### Determination of germination and greening

Wild-type WT, single mutant *lrr1-1*, *lrr1-2*, *kin7*, double mutant *lrr1kin7*, overexpression lines *LRR1ox2*, *LRR1ox10* seed materials of Arabidopsis were disinfected and then planted on MS medium plate (no sugar) containing ABA. The control group was MS medium plate (no sugar) without ABA. The seeds were refrigerated at 4 °C for 2–3 days. Then, they were cultured in the light culture room, The time calculation starts from the day of putting them into the light incubator, 24 h later is the first day, and the germination percentage was calculated with the index of two mm of seed radicle exposed, statistics were collected for six days. The cotyledons of the control material turned green in about seven days, and the cotyledon greening assay was counted from the seventh day.

### *pHBT-LRR-GFP* eukaryotic expression vector construction

The gene sequence of CDS was obtained from the *Arabidopsis thaliana* website (http://www.arabidopsis.org) using a primer with specific primers, primer 5.0 software design based on cDNA template, by PCR amplification of CDS length sequence. Target fragments were acquired through double enzyme digestion (*Nco*I and *Sal*I). The resulting product was ligated with *pHBT-GFP* plasmid, which had been cut with the same enzymes, at 16 °C. This ligation product was then transformed into *E. coli DH5α* recipient cells. Following overnight growth, colonies were subjected to colony PCR analysis to identify positive clones for further confirmation of recombinant plasmids *via* double enzyme digestion in plasmid PCR assays. The source of plasmids used in this experiment is from the Key Laboratory of Natural Products, Henan Academy of Sciences, China. All of the primers and cloning sites for vector construction are listed in Table S1.

### Construction of vectors with *LRR1* gene and plant transformation

The CDS sequence of *LRR1* was obtained from the Arabidopsis website (https://www.arabidopsis.org/), and targeted primers were designed using Primer 5.0 software. The full-length CDS sequence was amplified by PCR, utilizing cDNA as a template. The CDS sequence of *LRR1* was subjected to double digestion using *Sal*I and *Spe*I enzymes to obtain the target fragment. The target fragment was ligated with the same double-digested *pCAMBIA1300-GFP* plasmid at 16 °C, and the ligated product was transformed into *E. coli DH5α* receptor cells. The overnight-grown colonies were subjected to colony PCR, and the positive colonies were selected for plasmid PCR further to confirm the recombinant plasmid through double enzyme digestion. The CDS obtained by double enzyme digestion was recovered, and the sequencing-free plasmid was transformed into *Agrobacterium tumefaciens GV3101* receptor cells, which were identified by colony PCR and then infiltrated into Arabidopsis seedlings according to the Agrobacterium flower immersion method. After harvesting the F_1_ generation of seeds, the seeds were spotted on screening MS plates containing hygromycin, and the seeds were collected singly when the plants matured, repeating the screening process three times. Screening of pure and transgenic plants is still in progress. To complement *lrr1-1*, we amplified and cloned the *LRR1* coding region into the *pCAMBIASP*^+^*1300* vector to generate *35S:LRR1*. The constructs were transformed into *lrr1-1* to generate complemented transgenic plants. The *35S:LRR1* plasmids were also transformed into Col-0 to generate *LRR1*-overexpressing transgenic plants (*LRR1ox2*, *LRR1ox10*). For histochemical analysis of *LRR1*, we amplified approximately 1,000 bp promoter fragment of *LRR1* from Col-0 genomic DNA and then cloned it into beta-glucuronidase (GUS) expression vector *pCAMBIA1381* (Clontech). The *LRR1pro:GUS* construct was transformed into Col-0. The source of plasmids used in this experiment is from the Key Laboratory of Natural Products, Henan Academy of Sciences, China. All of the primers and cloning sites for vector construction are listed in Table S1.

### Tissue Localization of β-Glucuronidase

T3 transgenic seedlings or tissues were harvested and incubated in freshly prepared buffer containing 5-bromo-4-chloro-3-indolyl-β-D-glucuronic acid for 4 h at 37 °C in the dark, then washed with 70% ethanol. Images were observed by a stereo fluorescence microscope.

### RNA extraction and RT-PCR assay

Total RNA was extracted from seedlings by RNA extraction kit (Tiangen, Beijing, China). After DNase I treatment, reverse transcription was conducted using PrimeScript II 1st Strand cDNA Synthesis Kit (Tiangen) according to the manufacturer’s protocol.

Through agar-gel electrophoresis, the template amount of each sample was adjusted so that the product amount of *ACTIN2* obtained by amplification of different samples with the adjusted template amount was consistent. Then, the *LRR1* gene was amplified with the sample’s adjusted template amount to detect the target gene’s expression difference in different samples. The primers utilized in this study are listed in Table S2.

### qRT-PCR assay

*LRR1* cDNA template in each reaction tube was diluted 3–5 times, and 2 µL of diluted cDNA, SYBR premix EX Taq 10 µL, ROX dye (control dye) 0.4 µL, and upstream and downstream specific primers 0.4 µL (10 µM) were added. Then the reaction was carried out on a qRT-PCR instrument. According to the type of instrument used and the characteristics of the qRT-PCR kit used, the reaction procedure was set as pre-denaturation at 94 °C for 30 s; denaturation at 94 °C for 15 s, annealing at 55 °C for 20 s, 40 cycles of the above two steps; extension at 75 °C for 15 s, and analyzed according to software data after amplification. The amplification efficiency and Ct values were calculated using a program with at least three parallel reactions per sample, repeated three times. Standard computational methods were used to analyze gene expression. The primers utilized in this study are listed in Table S2 (qRT-PCR primer design: through www.ncbi.nlm.nih.gov/tools/primer-blast/).

### Yeast two-hybrid assay

Saccharomyces cerevisiae strain Y2HGold (Clontech, Mountain View, CA, USA) was used for co-transformation of the activation domain (AD) and binding domain (BD) constructs. *PYL1* to *PYL9*, *SnRK2.1* to *SnRK 2.10*, *ABI1*, and *ABI2* were cloned into the pGAD-T7 vector to generate the AD-ABI1, AD-ABI2, AD-SnRK(2.1∼2.10), AD-PYL(1∼9) plasmids, and the full-length sequences of *LRR1* and *KIN7* were cloned into pGBK-T7 to generate the BD-LRR1 and BD-KIN7 plasmids, respectively. A series of diluted co-transformed Y2H Gold cultures were spotted onto synthetic defined plates lacking Trp and Leu or lacking Trp, Leu, His, and Ade, and incubated at 30 °C for 3 days to observe yeast growth. All of the primers for plasmid construction are listed in Table S3.

### BiFC assay

The coding regions of *ABI1*, *ABI2*, and *PYL6* were cloned into the binary BiFC vector pSPYNE173 to produce the ABI1-YFP^N^, ABI2-YFP^N^, and PYL6-YFP^N^ plasmids, respectively. The coding regions of *LRR1* and *KIN7* were cloned into pSPYCE(M) to produce LRR1-YFP^C^ and KIN7-YFP^C^. Constructs including empty vectors were introduced into the Agrobacterium strain *GV3101* and then infiltrated into *Nicotiana benthamiana* leaves. YFP signals were observed using confocal microscopy 2 days after infiltration. All primers used for plasmid construction are listed in Table S3.

### GST pull-down assay

The coding region of *LRR1* was cloned into pGEX-4T-1 to obtain the LRR1-GST recombinant protein, and the coding region of *ABI1* and *PYL6* were cloned into pET28a to obtain the ABI1-His and PYL6-His recombinant protein. Glutathione beads containing LRR1-GST were incubated with ABI1-His and PYL6-His proteins in 1 ×PBS buffer at 4 °C for 2 h. The beads were washed five times with wash buffer (1 ×PBS, 0.1% Triton X-100). Proteins retained on the beads were analyzed by immunoblotting with an anti-GST or anti-His antibody. All primers used for plasmid construction are listed in Table S3.

## Results

### Expression pattern of *LRR1*

**Figure 1:**
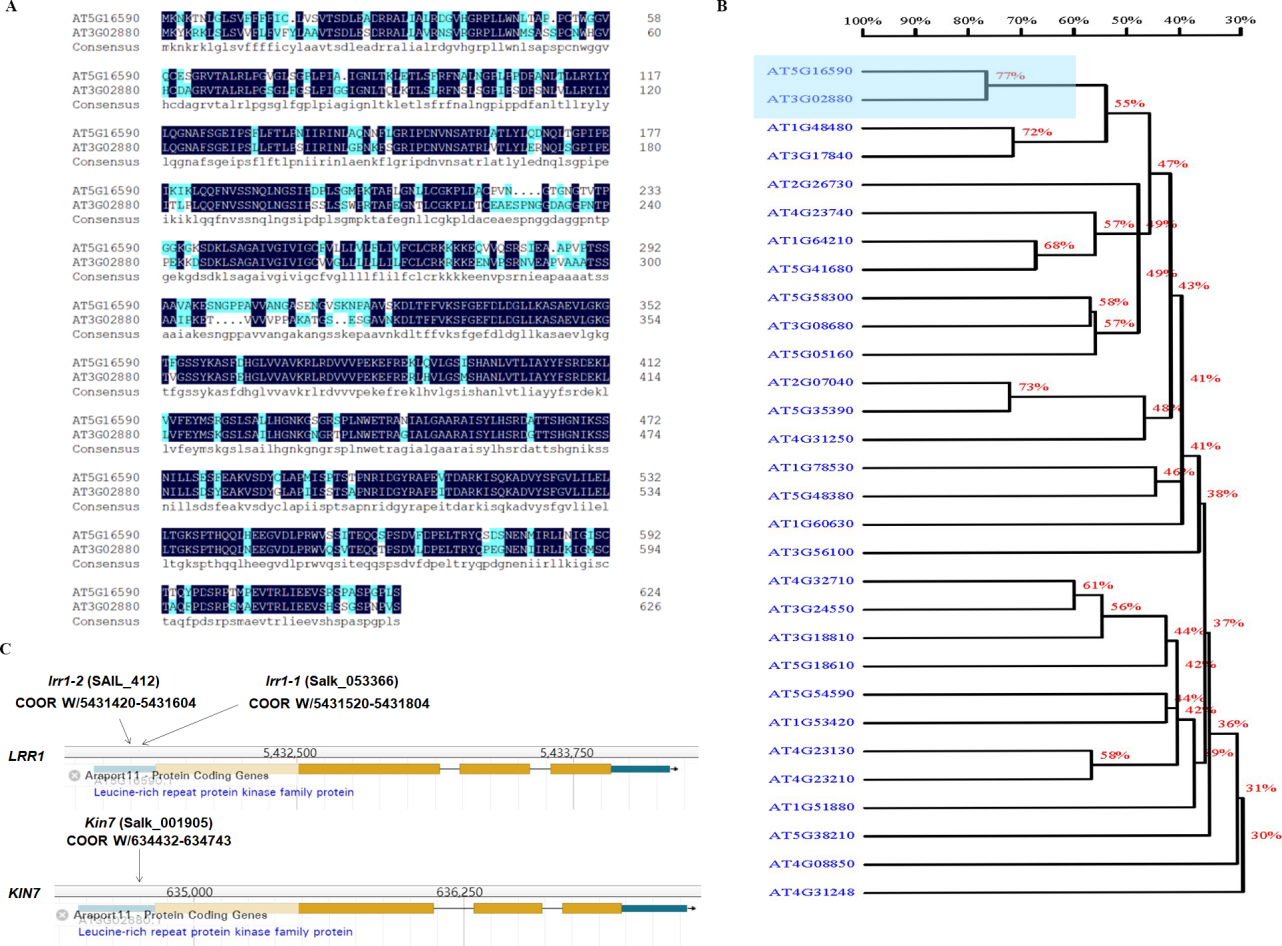
Correlation of *LRR1* with *KIN7*. (A) The protein sequences of LRR1 and KIN7 (480 bp downstream of ATG) were compared. (B) Phylogenetic tree of LRRs in Arabidopsis. The phylogenetic tree was generated using MEGA11. Full-length amino acid sequences of plant LRRs were selected for generating a bootstrap neighbor-joining phylogenetic tree. Bootstrap probabilities were obtained from 1000 replicates. A scale bar is indicated. (C) T-DNA insertion sites for *lrr1-1*, *lrr1-2*, and *kin7*. T-DNA was inserted near the promoter region 1000 bp upstream of the ATG, respectively.

ABA plays an important role in regulating plant growth and development. About 10% of genes in Arabidopsis are expressed by ABA induction, including various protein kinases (Baek, Lim & Lee, 2018). The cascades of LRR-RLK receptor protein kinases are involved in ABA-regulated plant growth and development. The analysis of the phylogenetic tree of LRR-RLKs in Arabidopsis showed that the protein sequences of *KIN7* and *LRR1* genes exhibited a relatively high degree of similarity (Figs. 1A & 1B). The results analyzed through the TAIR website show that the T-DNA of *lrr1-1*, *lrr1-2*, and *kin7* were inserted into the promoter region approximately 1,000 bp upstream of the start codon ATG (Fig. 1C). The *lrr1-2* mutant appears to produce a lower level of the RT-PCR band than WT, while the *lrr1-1* does not appear to have any band. In addition, the *kin7* allele also appears to produce some RNA. These results would seem to indicate that the *lrr1-2*, *kin7* mutants are not nulls, but down-regulated expression (Fig. S1).

**Figure 2:**
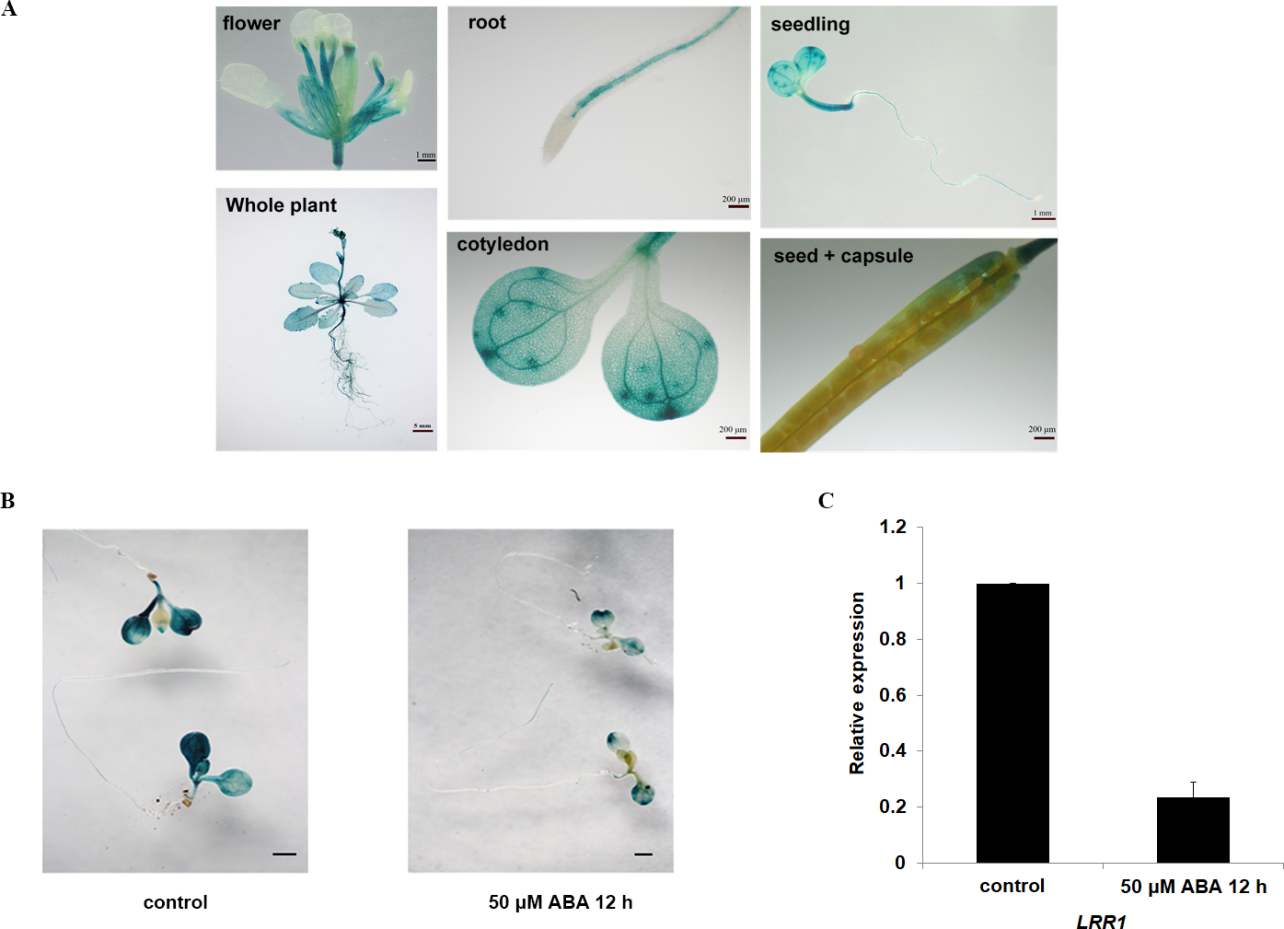
The identification and gene expression analysis. (A) Histochemical analysis of the GUS gene driven by the *LRR1* promoter. (B) ABA treatment of *LRR1-GUS* transgenic plants. Applying 50 µM ABA at 12 h, control: 0 µM ABA at 12 h. Cotyledon GUS staining gradually faded (Scale bar is one mm). (C) *LRR1* gene expression. qRT-PCR results of expression of *LRR1* gene in WT seedling treated with 50 µM ABA for 12 h. Control: materials 50 µM ABA at 0 h. The expression of LRR1 after ABA treatment is normalized compared to expression without ABA, which is set at a value of 1. Each value is the mean ± SD of three independent biological determinations.

The expression of the *GUS* gene driven by the *LRR1* promoter in various tissues and organs of Arabidopsis, as shown in Fig. 2A. Among them, the expression of the *LRR1* gene was abundant in roots, stems, and petioles. The results indicated that LRR1 is widely expressed in multiple organs. To verify the relationship between *LRR1* and ABA, RT-PCR was employed to assess the expression of the *LRR1* gene in each material treated with ABA (Fig. S2). Further, the GUS transgenic plants of *LRR1* were treated with ABA. GUS activity was lower in seedlings treated for with ABA for longer periods of time. The results showed that *LRR1* gene expression was gradually down-regulated with the increase in ABA treatment time (Fig. 2B). qRT-PCR assay: After exogenous ABA treatment, the expression level of *LRR1* was down-regulated (Fig. 2C).

### Transient expression and GFP fluorescence assay with *LRR1*

**Figure 3:**
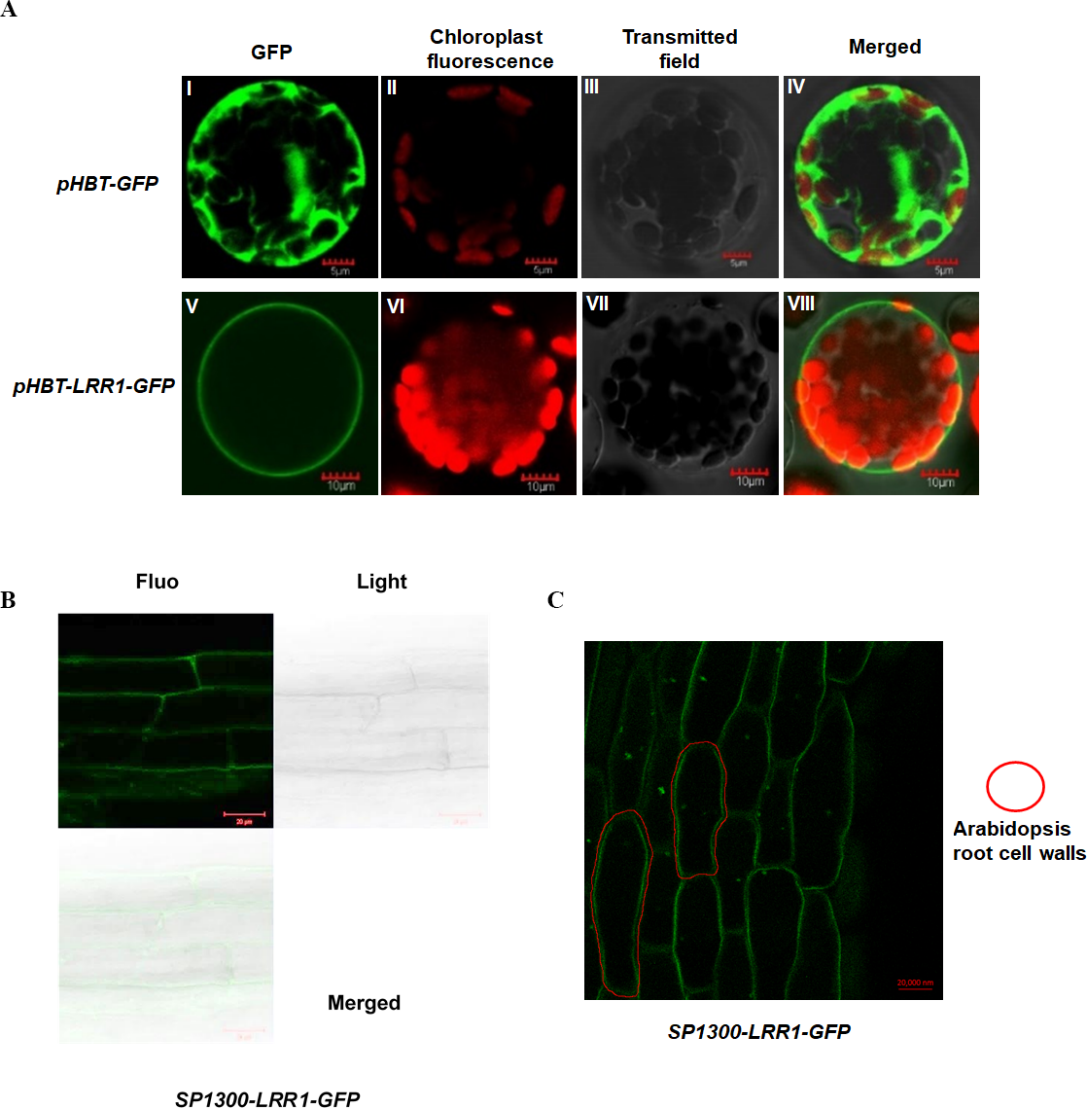
Subcellular localization of LRR1 on the cell membrane of transiently transformed protoplasts. (A) I: The GFP fluorescence of empty vector; II: The chloroplast fluorescence of Arabidopsis protoplast; III: The transmitted image of Arabidopsis protoplast; IV: The merged image of I, II and III; V: The GFP fluorescence signal of *pHBT-LRR1-GFP*; VI: The chloroplast fluorescence of Arabidopsis protoplast; VII: The transmitted image of Arabidopsis protoplast; VIII: The Merged image of V, VI, and VII. (B) Subcellular localization of LRR1 on the root cell membrane was determined by using a permanent *SP1300-LRR1-GFP* vector. (C) After dehydration, root cell experiments showed that LRR1 was located on the cell membrane.

To analyze the subcellular localization of *LRR1,* the vector *pHBT-LRR1-GFP* was constructed. After transient expression in Arabidopsis protoplasts, GFP alone is visible in the cytosol, but the LRR1-GFP fusion is detected only in the plasma membrane, suggesting that the endogenous LRR1 protein is protoplast membrane localized. We use confocal laser microscopy to detect the fluorescence of Arabidopsis leaf cell protoplasts (Fig. 3A).

To further analyze the subcellular localization of the LRR1 protein, and determine the expression and distribution of *LRR1* in Arabidopsis cells, we constructed the *SP1300-GFP* transgenic recombinant vector of *LRR1*. The fluorescence of Arabidopsis roots was detected by confocal (Fig. 3B). LRR1 may be located in the plasma membrane of Arabidopsis plants. Since plants have cell walls, to determine the location of LRR1 in the plasma membrane, the root material of Arabidopsis carrying *LRR1-GFP* transgenic recombinant vector was treated with 200 mM mannitol overnight (12 h) in this study, and the fluorescence of Arabidopsis roots was detected by confocal (Fig. 3C). LRR1 was located on the plasma membrane in Arabidopsis roots.

### Germination assay, growth conditions, ABA stress

### *LRR1* involved in the regulation of seed germination inhibited by ABA

**Figure 4:**
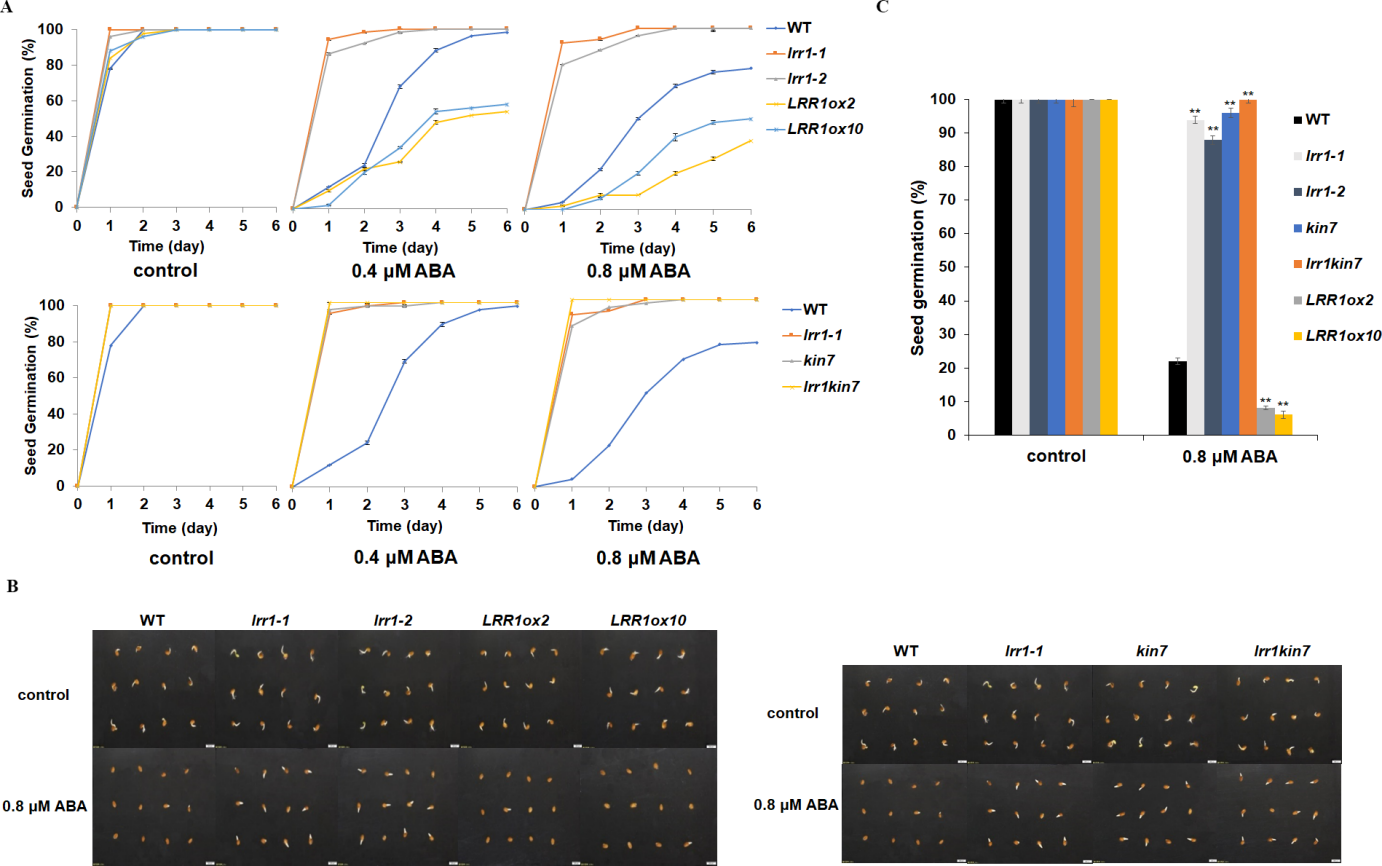
Participation of *LRR1* and *kin7* in the regulation of seed germination during the ABA response (In the case of low concentration ABA treatment). (A–C) The seed germination percentage of *lrr1-1*, *lrr1-2*, *kin7*, *lrr1kin7*, *LRR1ox2*, *LRR1ox10*, and wild-type WT in response to ABA; control: MS medium plate without ABA. (A) The effect of 0.4 M m, 0.8 µM ABA on seed germination was measured in 6 days. Values are the means of approximately 25 seeds (±SD) from three independent experiments. (B&C) Germination of seeds on MS medium (0.8 µM ABA) on the second day. Asterisks (**) indicate a significant difference compared with WT (Student’s *t*-test, *P* < 0.01), relevant and comprehensive data in Data S1.

LRR1 is a leucine-rich repeat receptor protein kinase, LRR-RLK, isolated from Arabidopsis. Its expression is down-regulated by dehydration, hypersaline, and low temperature (Soltabayeva et al., 2022). To determine the role of *LRR1* in ABA signaling, we analyzed the effects of ABA treatment on wild-type, single mutants, double mutant, and overexpression lines cultivated on MS medium plates with different concentrations of ABA, either 0.4 µM or 0.8 µM. The control was an MS medium plate without ABA. The seeds were refrigerated at 4 °C for 2–3 days. Then, they were cultured in the light culture room, The time calculation starts from the day of putting them into the light incubator, 24 h later is the first day, and the germination percentage was calculated with the index of two mm of seed radicle exposed, statistics were collected for 6 days. The germination percentage of wild-type WT decreased after treatment with 0.8 µM ABA (Fig. 4A). The germination percentage of mutants *lrr1-1*, *lrr1-2*, *kin7*, *lrr1kin7* and overexpression lines *LRR1ox2* and *LRR1ox10* without ABA treatment were the same. The germination percentage of mutants *lrr1-1*, *lrr1-2*, *kin7*, and *lrr1kin7* treated with 0.8 µM ABA showed difference from that treated with 0.4 µM ABA. Under 0.8 µM ABA treatment, the germination of *lrr1-1*, *lrr1-2*, *kin7*, and *lrr1kin7* was fast in the first 3 days (Figs. 4B & 4C).

**Figure 5:**
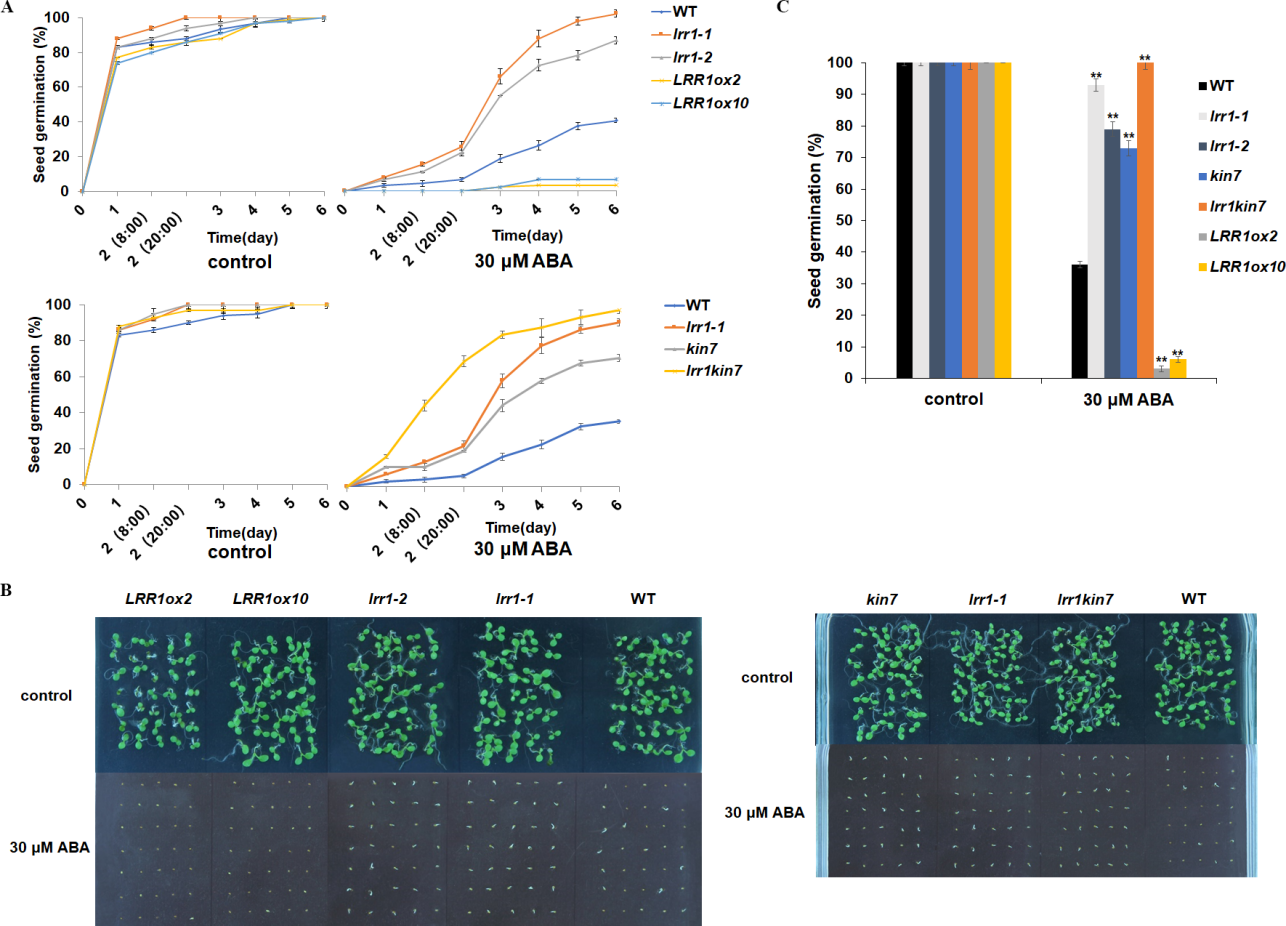
Participation of *LRR1* and *kin7* in the regulation of seed germination during the ABA response (In the case of high concentration ABA treatment). (A–C) The seed germination percentage of *lrr1-1*, *lrr1-2*, *kin7*, *lrr1kin7*, *LRR1ox2*, *LRR1ox10*, and wild-type WT in response to ABA; control: MS medium plate without ABA. (A) The effect of 30 µM ABA on seed germination was measured in 6 days. Values are the means of approximately 35 seeds (±SD) from three independent experiments. (B&C) Germination of seeds on MS medium (30 µM ABA) on the sixth day. Asterisks (**) indicate a significant difference compared with WT (Student’s *t*-test, *P* < 0.01), relevant and comprehensive data in Data S2.

Under 30 µM ABA treatment, the germination percentage of the wild-type was inhibited (Fig. 5A). The germination of double mutant *lrr1kin7* was fast in the first 3 days. In addition, the overexpression lines *LRR1ox2* and *LRR1ox10* were essentially non-germinating (Figs. 5B & 5C). In addition, the germination percentage of the mutant gradually slowed down with the extension of treatment time. These results suggest that *LRR1* gene knockout plants are less sensitive to ABA, and the *LRR1* gene may be involved in regulating the signal transduction process of ABA inhibition of seed germination.

### *LRR1* involved in ABA regulation of cotyledon greening

**Figure 6:**
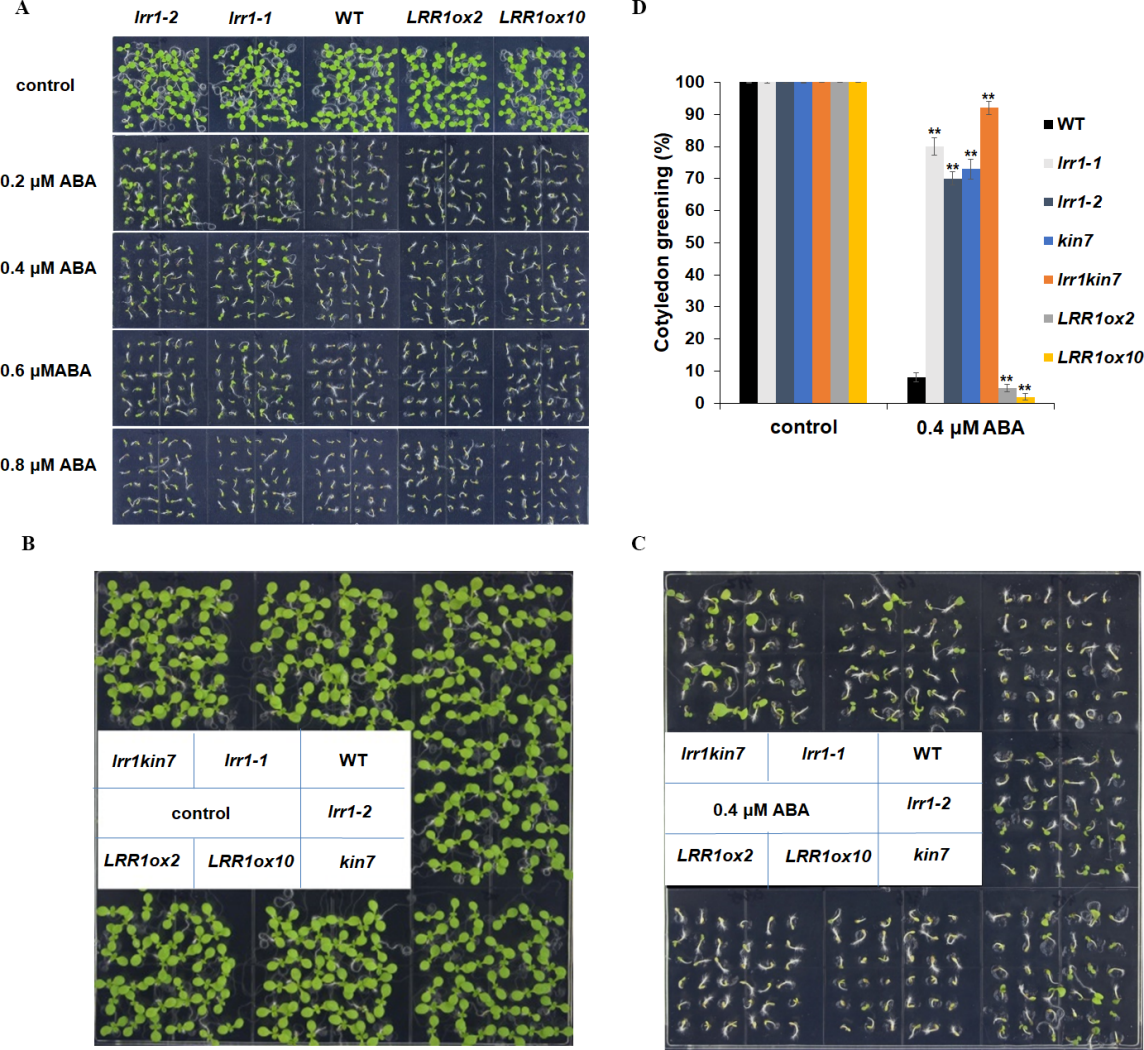
Cotyledon greening assay. (A) The effect of ABA (0.2 µM, 0.4 µM, 0.6 µM, 0.8 µM) on cotyledon greening; control: MS medium plate without ABA. Pictures were taken nine days later. (B & C) Cotyledons green of the wild type (WT), mutants (*lrr1-1*, *lrr1-2*, *kin7*, *lrr1kin7*), and overexpression lines (*LRR1ox2*, *LRR1ox10*). Seedlings were grown on 0.4 µM ABA MS medium (0.6% agar); control: MS medium plate without ABA , pictures were taken nine days later. (D) Comparison of cotyledon greening between the wild type, mutants, and overexpression lines in 9-d-old seedlings after 0.4 µM ABA treatment; control: 0 µM (±) ABA treatment nine days. Data bars represent means of cotyledons green from triplicate experiments (wild type and mutants, overexpression lines, *n* = 36), (±SD) from three independent experiments. Asterisks (**) indicate a significant difference compared with WT (Student’s *t*-test, *P* < 0.01), relevant and comprehensive data in Data S3.

We conducted the cotyledon greening test to validate the seed germination assay. This further elucidates that *LRR1* is involved in regulating the ABA signaling pathway. Notably, all cotyledons turned green without ABA in the MS medium. As ABA concentration increased, inhibition of cotyledon greening was observed across all materials tested. Interestingly, mutants *lrr1-1* and *lrr1-2* exhibited less inhibition while the overexpression lines exhibited greater inhibition when both are compared to wild-type. (Fig. 6A). This study suggests that the sensitivity of *LRR1* gene to ABA may implicate its involvement in regulating signal transduction processes associated with ABA-mediated inhibition of cotyledon greening.

This study further designed assays to conduct phenotypic analysis assays. The cotyledon of the materials in the MS medium without ABA had turned green, and the mutant materials *lrr1-1*, *lrr1-2*, and *kin7* after 0.4 µM ABA treatment had turned green with less inhibition compared with the wild-type WT material. The number of green cotyledons of double mutant *lrr1kin7* was more than that of single mutants *lrr1-1*, *lrr1-2*, and *kin7*. In contrast, the overexpression lines *LRR1ox2* and *LRR1ox10* were more susceptible to ABA inhibition than the wild-type, and the cotyledon did not turn green (Figs. 6B, 6C & 6D). The results of the above phenotyping experiments indicate that *LRR1* is involved in the ABA signaling pathway to regulate cotyledon greening.

### *LRR1* regulates ABA-induced gene expression

**Figure 7:**
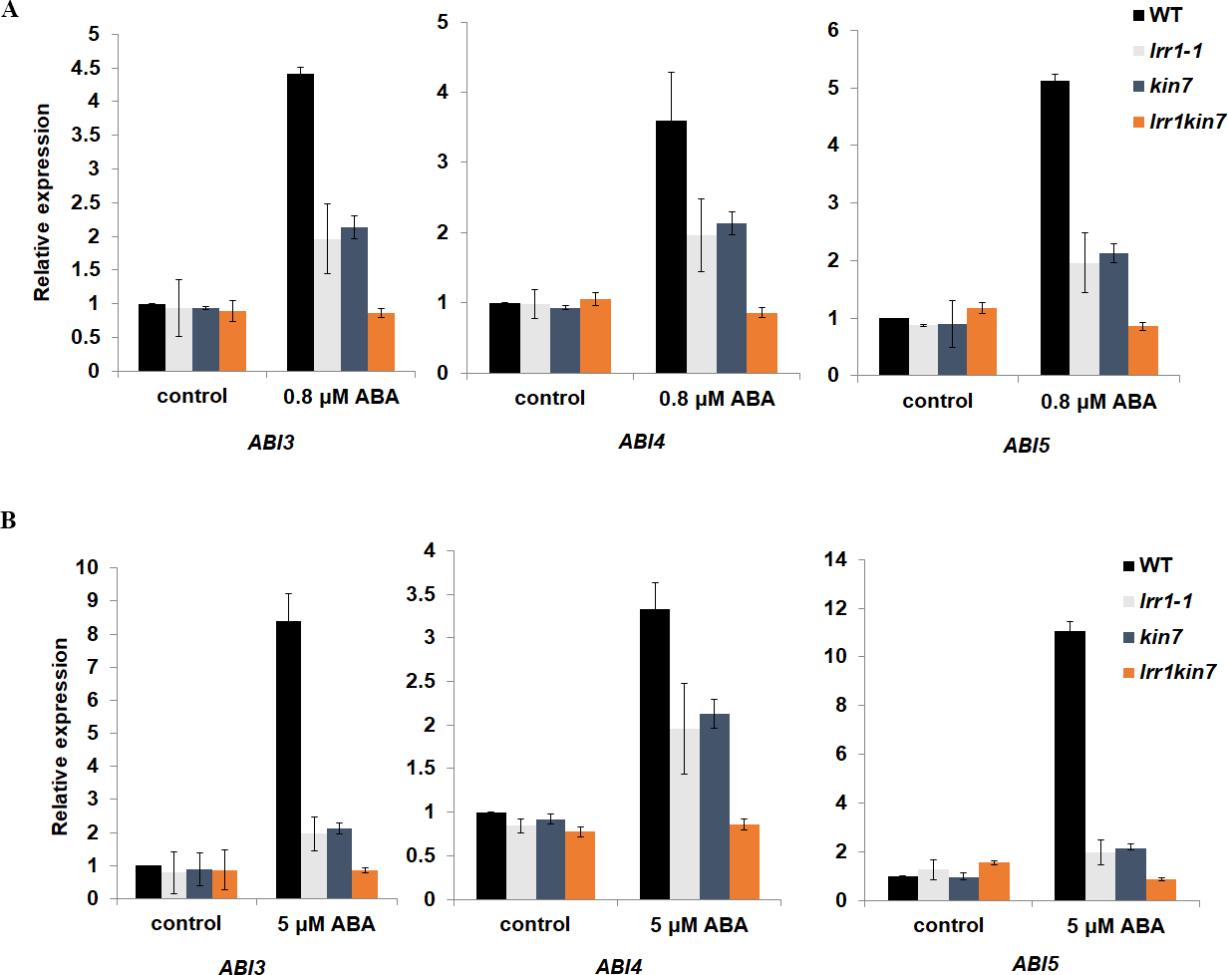
Expression of ABA-responsive genes was assayed by qRT-PCR in the wild-type WT and *LRR1*, *kin7*, *lrr1kin7*. (A) 1-day-old seedlings were treated with 0.8 µM (±) ABA for nine days. control: (0 µM (±) ABA, nine days). (B) Seeds treated with 5 µM (±) ABA for three days. control: 0 µM (±) ABA, three days. (A&B) Each value is the mean ± SD of three independent biological determinations, relevant and comprehensive data in Data S4–S9.

We have preliminarily demonstrated that *LRR1* is involved in the ABA signaling pathway to regulate seed germination and cotyledon greening during early development in Arabidopsis. To prove the function of *LRR1* in the ABA regulation of gene expression, qRT-PCR analysis was performed. The transcription factors ABI3, ABI4, and ABI5, which regulate early growth and development in Arabidopsis downstream in the ABA signaling pathway, were selected as the objects of qRT-PCR amplification. Seed and cotyledon of wild-type WT, single mutants *lrr1-1*, *kin7*, and double mutant *lrr1kin7* were selected as the target materials. Seed material was treated with 5 µM ABA for three days (the treatment ended on the 3rd day after the seeds began to appear white). cotyledon material was treated with 0.8 µM ABA treatment for nine days (from the beginning of seed whitening to the end of cotyledon green treatment, nine days). As shown in Figs. 7A & 7B, qRT-PCR detection showed that the expression levels of *ABI3*, *ABI4*, and *ABI5* genes were reduced in single mutants *lrr1-1* and *kin7* treated by ABA. The expression levels of *ABI3*, *ABI4*, and *ABI5* genes in double mutant *lrr1kin7* after ABA treatment were lower than those in single mutants *lrr1-1* and *kin7*. In addition, *ABI3*, *ABI4*, and *ABI5* transcription factors play important roles in regulating seed germination and growth and development in the ABA signaling pathway. If the expression level of *ABI5* is increased, the seed will be dormant; if the expression level is decreased, the seed will break dormancy and start germination, growth, and development. The *ABI5* expression levels of single mutants *lrr1-1* and *kin7* were lower than those of wild-type WT after ABA treatment, which is by the fact that the germination percentage and cotyledon greening of single mutants *lrr1-1* and *kin7* were higher than those of wild-type WT.

### Proteins binding to LRR1 were identified by *in vivo* and *in vitro* protein interaction assays

**Figure 8:**
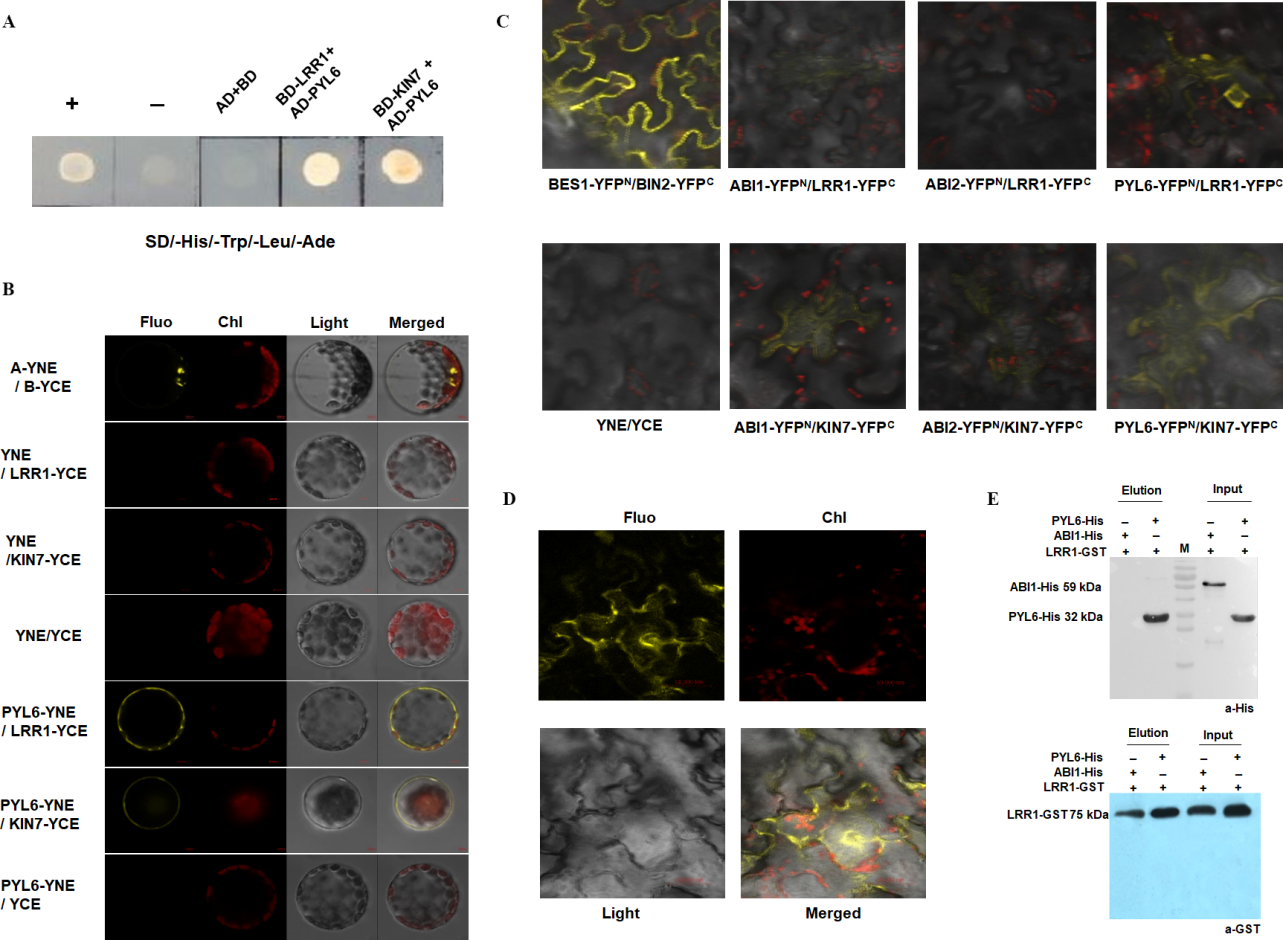
The interacting proteins of LRR1/KIN7 were screened by *in vitro* protein interaction assay. (A) Yeast two-hybrid complementation assay showed PYL6; PYL6 interacts with KIN7 (seed extraction). (B) BiFC assay (Arabidopsis) Bimolecular fluorescence experiments show PYL6-YNE/LRR1-YCE, PYL6-YNE/KIN7-YCE interaction. A-YNE/B-YCE were used as a control. (C) BiFC assay (Nicotiana) BiFC experiments showed that PYL6-YNE/LRR1-YCE interaction was observed through agrobacterium infection of tobacco. (D) BiFC assay (Nicotiana) BiFC experiments showed that PYL6-YNE/LRR1-YCE interaction was observed through agrobacterium infection of tobacco. (E) GST pull-down assay of LRR1 and PYL6. ABI1-His, PYL6-His, and LRR1-GST were expressed in *E. coli*. Purified proteins were used for the pull-down assay. ABI1-His and PYL6-His proteins were detected with anti-His antibodies, and LRR1-GST protein was detected with anti-GST antibodies. ABI1 protein was used as a control.

Previous assays have shown that *LRR1* and its homologous gene *KIN7* are involved in the ABA signaling pathway to regulate the growth and development of Arabidopsis seeds. However, the mechanism of the function of LRR1 and its homologous protein KIN7 in the ABA signaling pathway as well as the associated proteins, are unknown. To further screen the proteins associated with LRR1 and KIN7 in the ABA signaling pathway, we constructed a yeast two-hybrid vector, using LRR1-BD and KIN7-BD as bait proteins. Protein phosphatases ABI1 and ABI2 (repressors of ABA signaling pathway), protein kinases SnRK2.1 to SnRK2.10 and ABA receptors PYL1 to PYL9 were used as the target proteins (AD-ABI1, AD-ABI2, AD-SnRK(2.1∼2.10), and AD-PYL(1∼9)) to check their potential interaction with BD-LRR1 and BD-KIN7. we found that both BD-LRR1 and BD-KIN7 could interact with AD-PYL6 in yeast cells (Fig. 8A).

The GFP fluorescence of the transformed Arabidopsis protoplast was observed under a 60 × objective lens of a laser confocal scanning microscope. Fluorescence was found in the positive control group (Fig. 8B, top row), and LRR1-YCE and PYL6-YNE were observed. Both KIN7-YCE and PYL6-YNE showed fluorescence, while YNE and YCE were negative controls. YNE and LRR1-YCE, KIN7-YCE; Neither YCE nor PYL6-YNE fluoresces (Fig. 8B). The results show that LRR1 and PYL6 interact *in vivo*.

In addition, we used *Nicotiana benthamiana* leaves to verify the protein-protein interaction. The tobacco was injected at room temperature for 2 to 3 h. Two to five days after injection, the leaves of the tobacco were torn off, and the back of the leaves were taken under the 40 × objective lens of the laser confocal scanning microscope to observe the interaction fluorescence after transformation. Fluorescence was found in the positive control group, LRR1-YFP^C^, and PYL6- YFP^N^ were observed. KIN7-YFP^C^ and PYL6-YFP^N^ both fluoresce, while LRR1-YFP^C^ and PYL6-YFP^N^ fluoresce very strongly, and a specific group is made below. YNE and YCE were the negative controls. YNE and LRR1-YFP^C^, KIN7-YFP^C^; Neither YCE nor PYL6-YFP^N^ fluoresces (Figs. 8C & 8D). The assay of bimolecular fluorescence interaction between LRR1-YFP^C^ and PYL6- YFP^N^ is also consistent with the above assays. The experiment results show that LRR1 and PYL6 interact with each other.

To further detect the interaction between LRR1 and PYL6, we constructed prokaryotic expression vectors of LRR1-GST, PYL6-His, and ABI1-His. The protein interaction was demonstrated by the GST pull-down technique. PYL6-His was observed in the LRR1-GST pull-down and PYL6-His input control lanes (Fig. 8E). This implies that the interaction between LRR1 and PYL6 facilitates the retention of PYL6, as evidenced by its detection through Western Blot analysis. The assay results demonstrate an *in vitro* interaction between LRR1 and PYL6.

## Discussion

Abiotic stresses such as drought and high salinity profoundly impact plant growth, development, and global crop production. Since Arabidopsis and the oilseed crop Camelina sativa belong to the same family Cruciferae, and are closely related (Ghidoli et al., 2023). Therefore, this study selects *Arabidopsis thaliana* as a model plant to lay the foundation for subsequent related research, which has certain application value. While research on early growth and development in Arabidopsis lags behind that of above-ground plant parts, numerous studies have shown the combined regulation of early growth and development process by internal and external factors. Environmental factors like light, temperature, and humidity interact with hormones, sugars, nitrogen compounds, seed coat color, and structure to govern early growth and development in Arabidopsis. Notably, ABA plays a crucial role in regulating seed germination as well as subsequent stages of seedling growth.

The ABA signaling pathway, an important plant hormone, has been extensively studied and reported (Wang et al., 2020; Sano & Marion-Poll, 2021; Pan et al., 2021). Its growth-inhibiting function is recognized as a key factor in improving resilience to adversity (Brookbank et al., 2021; Yoshida et al., 2019b). However, since plant growth and stress adaptation processes are contradictory, it’s crucial to establish a balance between the two that suits the environment (Zhao et al., 2018a; Zhao et al., 2018b). Therefore, genetic regulation of ABA levels and/or signaling may yield useful crop varieties for specific environments to improve productivity. LRR-RLKs play a critical role in various aspects of plant growth and development by participating in signal recognition for ABA signal transduction regulation such as stomatal closure induced by ABA or H_2_O_2_ and inhibition of seed germination/post-germination growth by ABA. Additionally, the intricate nature of signal transduction between LRR-RLKs and ABA underscores the need for further exploration into their interplay during plant growth and development.

In this study, the transgenic *pCAMBIA1381-LRR1-GUS* seedlings subjected to ABA at different periods showed that the expression level of *LRR1* in transgenic seedlings treated with GUS staining was gradually weakened with time after ABA application, suggesting that *LRR1* was down-regulated by ABA. The RT-PCR and qRT-PCR also proved the above results (Figs. 2B & 2C) (Fig. S2). The findings of this study suggest that the regulation of *LRR1* may be influenced by ABA.

Mutants *lrr1-1* and *lrr1-2* with down-regulated *LRR1* gene expression were obtained by using the T-DNA insertion mutation technique. In the assay, we found that the mutation of the *LRR1* gene only led to the downregulation of its activity, which may be related to the following factors:

On the one hand, the *LRR1* mutant used in the assay was a mutant with downregulation of T-DNA insertion gene expression, and did not completely block the kinase activity of *LRR1*, suggesting a positive role in ABA signaling. We further constructed *LRR1* overexpression vectors *LRR1ox2* and *LRR1ox10* and the results confirmed the above ideas (Figs. 4–6). Because they are even more sensitive to ABA than WT.

On the other hand, bioinformatics analysis found that there is a protein with high homology of LRR1 amino acid sequence in Arabidopsis, that is *KIN7*. It has been reported that *KIN7* is involved in fine-tuning ABA-induced stomatal closure (Isner et al., 2018). Suggesting that *KIN7* and *LRR1* may be functionally redundant. For this purpose, we designed experiments, the mutation of *kin7* gene *KIN7* was obtained by using T-DNA insertion mutation technology. The double mutant *lrr1kin7* was constructed. Phenotypic analysis showed that *lrr1kin7* was less sensitive to ABA than *lrr1-1*, *lrr1-2*, and *kin7*. Because the phenotypes of the double mutant appear additive compared to each of the singles, that is, more severe than either single mutant, this result provides evidence that the proteins are partially functionally redundant in these processes.

In addition, the ABA-responsive genes *ABI3*, *ABI4*, and *ABI5* are crucial for regulating seed germination and cotyledon greening in early plant growth. High ABI3, ABI4, and ABI5 expression inhibit seed germination (Li et al., 2021). In this study, we selected these genes as targets for qRT-PCR analysis. significant downregulation was observed after ABA treatment in the expression of these genes in mutants (*lrr1-1*, *kin7*) compared to WT in Arabidopsis seeds and leaf tissues. These findings suggest that LRR1 regulates the ABA signaling pathway by modulating downstream transcription factors such as *ABI3*, *ABI4*, and *ABI5*. Physiological phenotypic assays revealed that mutant materials (*lrr1-1*, *kin7*) treated with ABA showed unaffected seed germination and cotyledon greening compared to wild-type plants which exhibited impaired growth in both seeds and cotyledons. Therefore, it can be concluded that *LRR1* is involved in the accumulation process of *ABI3*, *ABI4*, and *ABI5*; downregulation of *LRR1* expression leads to decreased levels of these genes resulting in breaking seed dormancy and initiating germination and seedling growth. These results confirmed that *LRR1* may be involved in the regulation of early growth and development in Arabidopsis by positively affecting ABA response.

PYL receptors in the ABA signaling pathway function as key upstream regulators. Their ability to bind ABA, interact with PP2Cs, and activate SnRK2 kinases underscores their crucial role in ABA signaling. In this study, the interaction between LRR1 and PYL6 in the ABA signaling pathway was detected by yeast two-hybrid assay, bimolecular fluorescence complementation, and GST pull-down assays. The results indicated that PYL6 may be the substrate of LRR1 phosphorylation activation. Thus, LRR1 is potentially involved in the regulation of the ABA signaling pathway, thereby influencing the early growth and development of Arabidopsis through its interaction with PYL6.

PYL6 protein is classified as receptor protein involved in the reception of exogenous signals, with PYL6 widely recognized as an ABA receptor. This study provides evidence suggesting that the involvement of the LRR1 protein in the ABA signaling pathway regulates early growth and development in Arabidopsis. However, further investigation is required to elucidate the interaction between LRR1 and PYL6 proteins and their role in regulating Arabidopsis’ early growth and development.

## Conclusion

Arabidopsis was employed as the experimental model in this investigation, wherein it was discovered that *LRR1* exerts a positive regulatory role in the early growth and developmental processes of Arabidopsis, including ABA-mediated inhibition of seed germination and cotyledon greening. Using molecular genetics, phenotypic analysis of the mutant with T-DNA insertion showed that *LRR1* positively regulates the effect of the ABA signaling pathway on early growth and development of Arabidopsis, such as seed germination and cotyledon greening. The downregulation of the LRR1 gene decreased Arabidopsis sensitivity to ABA. It is localized in the cell membrane and expressed in roots, stems, flowers, leaves, seeds, fruit pods, and hypocotyl. Through RNA transcriptomics and qRT-PCR analysis, it was found that the transcription factor *ABI5* may be involved in the regulation of seed germination and cotyledon greening of *LRR1* in response to ABA. By biochemical means, *in vitro* protein interaction assays, including bimolecular fluorescence complementarity assay, yeast two-hybrid complementarity assay, and GST pull-down assay, showed that LRR1 could interact with PYL6.

In summary, we found that *LRR1* is involved in the ABA signaling pathway to regulate the early growth and development of *Arabidopsis*.

## Supplemental Information

10.7717/peerj.18460/supp-1 Supplemental Information 1 Germination assay with 30 µM treatment

10.7717/peerj.18460/supp-2 Supplemental Information 2 Germination assay with 0.4 µM, 0.8 µMABA treatment

10.7717/peerj.18460/supp-3 Supplemental Information 3 Cotyledon greening assay

10.7717/peerj.18460/supp-4 Supplemental Information 4 LRR1 regulates ABA-induced gene expression data, 0.8 µM (±) ABA treatment ABI3

10.7717/peerj.18460/supp-5 Supplemental Information 5 LRR1 regulates ABA-induced gene expression data, 5 µM (±) ABA treatment ABI3

10.7717/peerj.18460/supp-6 Supplemental Information 6 LRR1 regulates ABA-induced gene expression data, 0.8 µM (±) ABA treatment ABI4

10.7717/peerj.18460/supp-7 Supplemental Information 7 LRR1 regulates ABA-induced gene expression data, 5 µM (±) ABA treatment ABI4

10.7717/peerj.18460/supp-8 Supplemental Information 8 LRR1 regulates ABA-induced gene expression data, 0.8 µM (±) ABA treatment ABI5

10.7717/peerj.18460/supp-9 Supplemental Information 9 LRR1 regulates ABA-induced gene expression data, 5 µM (±) ABA treatment ABI5

10.7717/peerj.18460/supp-10 Supplemental Information 10 Identification and characterization of T-DNA insertions in the *LRR1* and *KIN7* genes, along with gene expression analysis using semi-quantitative assays (A) T-DNA insertion sites for *lrr1-1*, *lrr1-2*, and *kin7*. T-DNA was inserted near the promoter region 1000 bp upstream of the ATG, respectively. (B) Mutants *lrr1-2* using LP+RP/LB1+RP as primers. *lrr1-2* (SAIL_412, LP:5′CCATGAAATAAAAGGGGCTTC3′, RP: 5′GACTCGGAGATTGGTTTTTCC3′, LB1:5′TCAAACAGGATTTTCGCCTGCT3′). (C) Double mutant *lrr1 kin7* using LP+RP/LBa1+RP as primers. *lrr1-1* (Salk_053366, LP: 5′GCTGGGGGTAAAGAATGAGAC3′, RP:5′ATTCTTCGTCTCCTTGGTTCC3′, LBa1:5′ TGGTTCACGTAGTGGGCCATCG3′), *kin7* (Salk_001905, LP: 5′GCTGGGGGTAAAGAATGAGAC3′, RP:5′ATTCTTCGTCTCCTTGGTTCC3′, LBa1:5′ TGGTTCACGTAGTGGGCCATCG3′), M: DNA Marker(100-2000bp). (D) RT-PCR results of expression of *LRR1* gene in wild-type WT, mutants *lrr1-1* and *lrr1-2* , as well as Overexpressed material s *LRR1ox2* and *LRR1ox10*. (E) RT-PCR results of expression of *KIN7* gene in wild type WT, mutant *kin7* . (D&E) RT-PCR assays showed that the expression of T-DNA mutants of *LRR1* and *KIN7* genes was not completely downregulated.

10.7717/peerj.18460/supp-11 Supplemental Information 11 RT-PCR was employed to assess the_expression of LRR1 gene in each material treated with ABA shows the RT-PCR results of the expression of the *LRR1* gene in wild-type WT mutants *lrr1-1* Overexpressed materials *LRR1ox2* and *LRR1ox10* were treated with 50 µM ABA for 12 h.

10.7717/peerj.18460/supp-12 Supplemental Information 12 PCR primers were commonly used in the experiments

10.7717/peerj.18460/supp-13 Supplemental Information 13 Primer sets for conducting RT-PCR assay, qRT-PCR assay

10.7717/peerj.18460/supp-14 Supplemental Information 14 Primer sets for conducting yeast two-hybrid assay, BiFC assay and GST Pull-Down assay

## Additional Information and Declarations

### DNA Deposition

The following information was supplied regarding the deposition of DNA sequences: The LRB1 and LRB2 sequences are available at TAIR: AT5g16590–AT3g02880.
https://www.arabidopsis.org/locus?key=134655

https://www.arabidopsis.org/locus?key=35803

### Data Availability

The following information was supplied regarding data availability: The raw data are available in the Supplemental Files.

## References

[ref-1] Ali A, Kim JK, Jan M, Khan HA, Khan IU, Shen M, Park J, Lim CJ, Hussain S, Baek D, Wang K, Chung WS, Rubio V, Lee SY, Gong Z, Kim WY, Bressan RA, Pardo JM, Yun DJ (2019). Rheostatic control of ABA signaling through HOS15-mediated OST1 degradation. Molecular Plant.

[ref-2] Baek W, Lim CW, Lee SC (2018). A DEAD-box RNA helicase, RH8, is critical for regulation of ABA signaling and the drought stress response via inhibition of PP2CA activity. Plant, Cell and Environment.

[ref-3] Becraft PW (2002). Receptor kinase signaling in plant development. Annual Review of Cell and Developmental Biology.

[ref-4] Brookbank BP, Patel J, Gazzarrini S, Nambara E (2021). Role of basal ABA in plant growth and development. Genes (Basel).

[ref-5] Chen K, Li GJ, Bressan RA, Song CP, Zhu JK, Zhao Y (2020). Abscisic acid dynamics, signaling, and functions in plants. Journal of Integrative Plant Biology.

[ref-6] Chen X, Wang T, Rehman AU, Wang Y, Qi J, Li Z, Song C, Wang B, Yang S, Gong Z (2021). Arabidopsis U-box E3 ubiquitin ligase PUB11 negatively regulates drought tolerance by degrading the receptor-like protein kinases LRR1 and KIN7. Journal of Integrative Plant Biology.

[ref-7] Collin A, Daszkowska-Golec A, Szarejko I (2021). Updates on the role of ABSCISIC ACID INSENSITIVE 5 (ABI5) and ABSCISIC ACID-RESPONSIVE ELEMENT BINDING FACTORs (ABFs) in ABA signaling in different developmental stages in plants. Cell.

[ref-8] Daszkowska-Golec A, Szarejko I (2013). Open or close the gate—stomata action under the control of phytohormones in drought stress conditions. Frontiers in Plant Science.

[ref-9] Dufayard JF, Bettembourg M, Fischer I, Droc G, Guiderdoni E, Périn C, Chantret N, Diévart A (2017). New insights on leucine-rich repeats receptor-like kinase orthologous relationships in angiosperms. Frontiers in Plant Science.

[ref-10] Emenecker RJ, Strader LC (2020). Auxin-abscisic acid interactions in plant growth and development. Biomolecules.

[ref-11] Fan M, Ma W, Liu C, Zhang C, Wu S, Chen M, Liu K, Cai F, Lin F (2018). Evolution and expression characteristics of receptor-like cytoplasmic protein kinases in maize, rice and arabidopsis. International Journal of Molecular Sciences.

[ref-12] Fiesselmann BS, Luichtl M, Yang X, Matthes M, Peis O, Torres-Ruiz RA (2015). Ectopic shoot meristem generation in monocotyledonous RPK1 mutants is linked to SAM loss and altered seedling morphology. BMC Plant Biology.

[ref-13] Fischer I, Diévart A, Droc G, Dufayard JF, Chantret N (2016). Evolutionary dynamics of the leucine-rich repeat receptor-like kinase (LRR-RLK) subfamily in angiosperms. Plant Physiology.

[ref-14] Fujita Y, Fujita M, Shinozaki K, Yamaguchi-Shinozaki K (2011). ABA-mediated transcriptional regulation in response to osmotic stress in plants. Journal of Plant Research.

[ref-15] Ghidoli M, Ponzoni E, Araniti F, Miglio D, Pilu R (2023). Genetic improvement of *Camelina sativa* (L.) crantz: opportunities and challenges. Plants (Basel).

[ref-16] Guo H, Nolan TM, Song G, Liu S, Xie Z, Chen J, Schnable PS, Walley JW, Yin Y (2018). FERONIA receptor kinase contributes to plant immunity by suppressing jasmonic acid signaling in arabidopsis. Current Biology.

[ref-17] He Y, Zhou J, Shan L, Meng X (2018). Plant cell surface receptor-mediated signaling—a common theme amid diversity. Journal of Cell Science.

[ref-18] Hohmann U, Santiago J, Nicolet J, Olsson V, Spiga FM, Hothorn LA, Butenko MA, Hothorn M (2018). Mechanistic basis for the activation of plant membrane receptor kinases by SERK-family coreceptors. Proceedings of the National Academy of Sciences of the United States of America.

[ref-19] Jaillais Y, Vert G (2016). Brassinosteroid signaling and BRI1 dynamics went underground. Current Opinion in Plant Biology.

[ref-20] Jamieson PA, Shan L, He P (2018). Plant cell surface molecular cypher: receptor-like proteins and their roles in immunity and development. Plant Science.

[ref-21] Je BI, Xu F, Wu Q, Liu L, Meeley R, Gallagher JP, Corcilius L, Payne RJ, Bartlett ME, Jackson D (2018). The CLAVATA receptor FASCIATED EAR2 responds to distinct CLE peptides by signaling through two downstream effectors. Elife.

[ref-22] Jia P, Xing L, Zhang C, Zhang D, Ma J, Zhao C, Han M, Ren X, An N (2021). MdKNOX19, a class II knotted-like transcription factor of apple, plays roles in ABA signaling/sensitivity by targeting ABI5 during organ development. Plant Science.

[ref-23] Lee IC, Hong SW, Whang SS, Lim PO, Nam HG, Koo JC (2011). Age-dependent action of an ABA-inducible receptor kinase, RPK1, as a positive regulator of senescence in Arabidopsis leaves. Plant and Cell Physiology.

[ref-24] Leung J, Merlot S, Giraudat J (1997). The arabidopsis ABSCISIC ACID-INSENSITIVE2 (ABI2) and ABI1 genes encode homologous protein phosphatases 2C involved in abscisic acid signal transduction. The Plant Cell.

[ref-25] Li C, Zhang S, Wang X (2017). Novel signaling interface constituted with membrane receptor-like kinases emerged from the study of interaction and transphosphorylation of BRI1 and BAK1. Current Topics in Medicinal Chemistry.

[ref-26] Li X, Zhong M, Qu L, Yang J, Liu X, Zhao Q, Liu X, Zhao X (2021). AtMYB32 regulates the ABA response by targeting ABI3, ABI4 and ABI5 and the drought response by targeting CBF4 in Arabidopsis. Plant Science.

[ref-27] Li L, Zhu T, Song Y, Feng L, Farag EAH, Ren M (2020). ABSCISIC ACID INSENSITIVE5 interacts with RIBOSOMAL S6 KINASE2 to mediate ABA responses during seedling growth in arabidopsis. Frontiers in Plant Science.

[ref-28] Liu PL, Du L, Huang Y, Gao SM, Yu M (2017). Origin and diversification of leucine-rich repeat receptor-like protein kinase (LRR-RLK) genes in plants. BMC Evolutionary Biology.

[ref-29] Liu YH, Jiang M, Li RQ, Huang JZ, Shu QY (2021). OsKEAP1 interacts with OsABI5 and its downregulation increases the transcription of OsABI5 and the ABA response genes in germinating rice seeds. Plants (Basel).

[ref-30] Lozano-Elena F, Caño Delgado AI (2019). Emerging roles of vascular brassinosteroid receptors of the BRI1-like family. Current Opinion in Plant Biology.

[ref-31] Ma Y, Szostkiewicz I, Korte A, Moes D, Yang Y, Christmann A, Grill E (2009). Regulators of PP2C phosphatase activity function as abscisic acid sensors. Science.

[ref-32] Man J, Gallagher JP, Bartlett M (2020). Structural evolution drives diversification of the large LRR-RLK gene family. New Phytologist.

[ref-33] Nagar P, Sharma N, Jain M, Sharma G, Prasad M, Mustafiz A (2022). OsPSKR15, a phytosulfokine receptor from rice enhances abscisic acid response and drought stress tolerance. Physiologia Plantarum.

[ref-34] Nakashima K, Fujita Y, Kanamori N, Katagiri T, Umezawa T, Kidokoro S, Maruyama K, Yoshida T, Ishiyama K, Kobayashi M, Shinozaki K, Yamaguchi-Shinozaki K (2009). Three Arabidopsis SnRK2 protein kinases, SRK2D/SnRK2.2, SRK2E/SnRK2.6/ OST1 and SRK2I/SnRK2.3, involved in ABA signaling are essential for the control of seed development and dormancy. Plant and Cell Physiology.

[ref-35] Nguyen QTC, Lee SJ, Choi SW, Na YJ, Song MR, Hoang QTN, Sim SY, Kim MS, Kim JI, Soh MS, Kim SY (2019). Arabidopsis raf-like kinase Raf10 is a regulatory component of core ABA signaling. Molecular Cell.

[ref-36] Osakabe Y, Mizuno S, Tanaka H, Maruyama K, Osakabe K, Todaka D, Fujita Y, Kobayashi M, Shinozaki K, Yamaguchi-Shinozaki K (2010). Overproduction of the membrane-bound receptor-like protein kinase 1, RPK1, enhances abiotic stress tolerance in arabidopsis. Journal of Biological Chemistry.

[ref-37] Pan J, Hu Y, Wang H, Guo Q, Chen Y, Howe GA, Yu D (2020). Molecular mechanism underlying the synergetic effect of jasmonate on abscisic acid signaling during seed germination in arabidopsis. The Plant Cell.

[ref-38] Pan W, Liang J, Sui J, Li J, Liu C, Xin Y, Zhang Y, Wang S, Zhao Y, Zhang J, Yi M, Gazzarrini S, Wu J (2021). ABA and bud dormancy in perennials: current knowledge and future perspective. Genes (Basel).

[ref-39] Park SY, Fung P, Nishimura N, Jensen DR, Fujii H, Zhao Y, Lumba S, Santiago J, Rodrigues A, Chow TF, Alfred SE, Bonetta D, Finkelstein R, Provart NJ, Desveaux D, Rodriguez PL, McCourt P, Zhu JK, Schroeder JI, Volkman BF, Cutler SR (2009). Abscisic acid inhibits type 2C protein phosphatases via the PYR/PYL family of START proteins. Science.

[ref-40] Peng J, Wang M, Wang X, Qi L, Guo C, Li H, Li C, Yan Y, Zhou Y, Terzaghi W, Li Z, Song CP, Qin F, Gong Z, Li J (2022). COP1 positively regulates ABA signaling during Arabidopsis seedling growth in darkness by mediating ABA-induced ABI5 accumulation. The Plant Cell.

[ref-41] Rahim AA, Uzair M, Rehman N, Rehman OU, Zahra N, Khan MR (2022). Genome-wide identification and characterization of receptor-like protein kinase 1 (RPK1) gene family in Triticum aestivum under drought stress. Frontiers in Genetics.

[ref-42] Samakovli D, Roka L, Plitsi PK, Drakakaki G, Haralampidis K, Stravopodis DJ, Hatzopoulos P, Milioni D (2022). BRI1 and BAK1 canonical distribution in plasma membrane is HSP90 dependent. Cell.

[ref-43] Sano N, Marion-Poll A (2021). ABA metabolism and homeostasis in seed dormancy and germination. International Journal of Molecular Sciences.

[ref-44] Santiago J, Dupeux F, Round A, Antoni R, Park SY, Jamin M, Cutler SR, Rodriguez PL, Márquez JA (2009). The abscisic acid receptor PYR1 in complex with abscisic acid. Nature.

[ref-45] Shang Y, Yang D, Ha Y, Shin HY, Nam KH (2020). Receptor-like protein kinases RPK1 and BAK1 sequentially form complexes with the cytoplasmic kinase OST1 to regulate ABA-induced stomatal closure. Journal of Experimental Botany.

[ref-46] Shimotohno A, Aki SS, Takahashi N, Umeda M (2021). Regulation of the plant cell cycle in response to hormones and the environment. Annual Review of Plant Biology.

[ref-47] Smakowska-Luzan E, Mott GA, Parys K, Stegmann M, Howton TC, Layeghifard M, Neuhold J, Lehner A, Kong J, Grünwald K, Weinberger N, Satbhai SB, Mayer D, Busch W, Madalinski M, Stolt-Bergner P, Provart NJ, Mukhtar MS, Zipfel C, Desveaux D, Guttman DS, Belkhadir Y (2018). An extracellular network of arabidopsis leucine-rich repeat receptor kinases. Nature.

[ref-48] Soltabayeva A, Dauletova N, Serik S, Sandybek M, Omondi JO, Kurmanbayeva A, Srivastava S (2022). Receptor-like kinases (LRR-RLKs) in response of plants to biotic and abiotic stresses. Plants (Basel).

[ref-49] Utsugi S, Ashikawa I, Nakamura S, Shibasaka M (2020). TaABI5, a wheat homolog of Arabidopsis ABA insensitive 5, controls seed germination. Journal of Plant Research.

[ref-50] Wang K, He J, Zhao Y, Wu T, Zhou X, Ding Y, Kong L, Wang X, Wang Y, Li J, Song CP, Wang B, Yang S, Zhu JK, Gong Z (2018). EAR1 negatively regulates ABA signaling by enhancing 2C protein phosphatase activity. The Plant Cell.

[ref-51] Wang Z, Ren Z, Cheng C, Wang T, Ji H, Zhao Y, Deng Z, Zhi L, Lu J, Wu X, Xu S, Cao M, Zhao H, Liu L, Zhu J, Li X (2020). Counteraction of ABA-mediated inhibition of seed germination and seedling establishment by ABA signaling terminator in arabidopsis. Molecular Plant.

[ref-52] Yoshida T, Christmann A, Yamaguchi-Shinozaki K, Grill E, Fernie AR (2019a). Revisiting the basal role of ABA—roles outside of stress. Trends in Plant Science.

[ref-53] Yoshida T, Obata T, Feil R, Lunn JE, Fujita Y, Yamaguchi-Shinozaki K, Fernie AR (2019b). The role of abscisic acid signaling in maintaining the metabolic balance required for arabidopsis growth under nonstress conditions. The Plant Cell.

[ref-54] Zhang H, Liu D, Yang B, Liu WZ, Mu B, Song H, Chen B, Li Y, Ren D, Deng H, Jiang YQ (2020). Arabidopsis CPK6 positively regulates ABA signaling and drought tolerance through phosphorylating ABA-responsive element-binding factors. Journal of Experimental Botany.

[ref-55] Zhao H, Nie K, Zhou H, Yan X, Zhan Q, Zheng Y, Song CP (2020). ABI5 modulates seed germination via feedback regulation of the expression of the PYR/PYL/RCAR ABA receptor genes. New Phytologist.

[ref-56] Zhao C, Zayed O, Yu Z, Jiang W, Zhu P, Hsu CC, Zhang L, Tao WA, Lozano-Durán R, Zhu JK (2018a). Leucine-rich repeat extensin proteins regulate plant salt tolerance in Arabidopsis. Proceedings of the National Academy of Sciences of the United States of America.

[ref-57] Zhao Y, Zhang Z, Gao J, Wang P, Hu T, Wang Z, Hou YJ, Wan Y, Liu W, Xie S, Lu T, Xue L, Liu Y, Macho AP, Tao WA, Bressan RA, Zhu JK (2018b). Arabidopsis duodecuple mutant of PYL ABA receptors reveals PYL repression of ABA-independent SnRK2 activity. Cell Reports.

[ref-58] Zhu Q, Feng Y, Xue J, Chen P, Zhang A, Yu Y (2023). Advances in receptor-like protein kinases in balancing plant growth and stress responses. Plants (Basel).

[ref-59] Zhu Y, Huang P, Guo P, Chong L, Yu G, Sun X, Hu T, Li Y, Hsu CC, Tang K, Zhou Y, Zhao C, Gao W, Tao WA, Mengiste T, Zhu JK (2020). CDK8 is associated with RAP2.6 and SnRK2.6 and positively modulates abscisic acid signaling and drought response in Arabidopsis. New Phytologist.

