# Supplementary figures and images for: LRR1 involved in the abscisic acid signaling pathway to regulate the early growth and development of Arabidopsis thaliana
Source: PeerJ. 2024 Nov 26;12:e18460. doi: 10.7717/peerj.18460 (PMC11606324; doi:10.7717/peerj.18460)

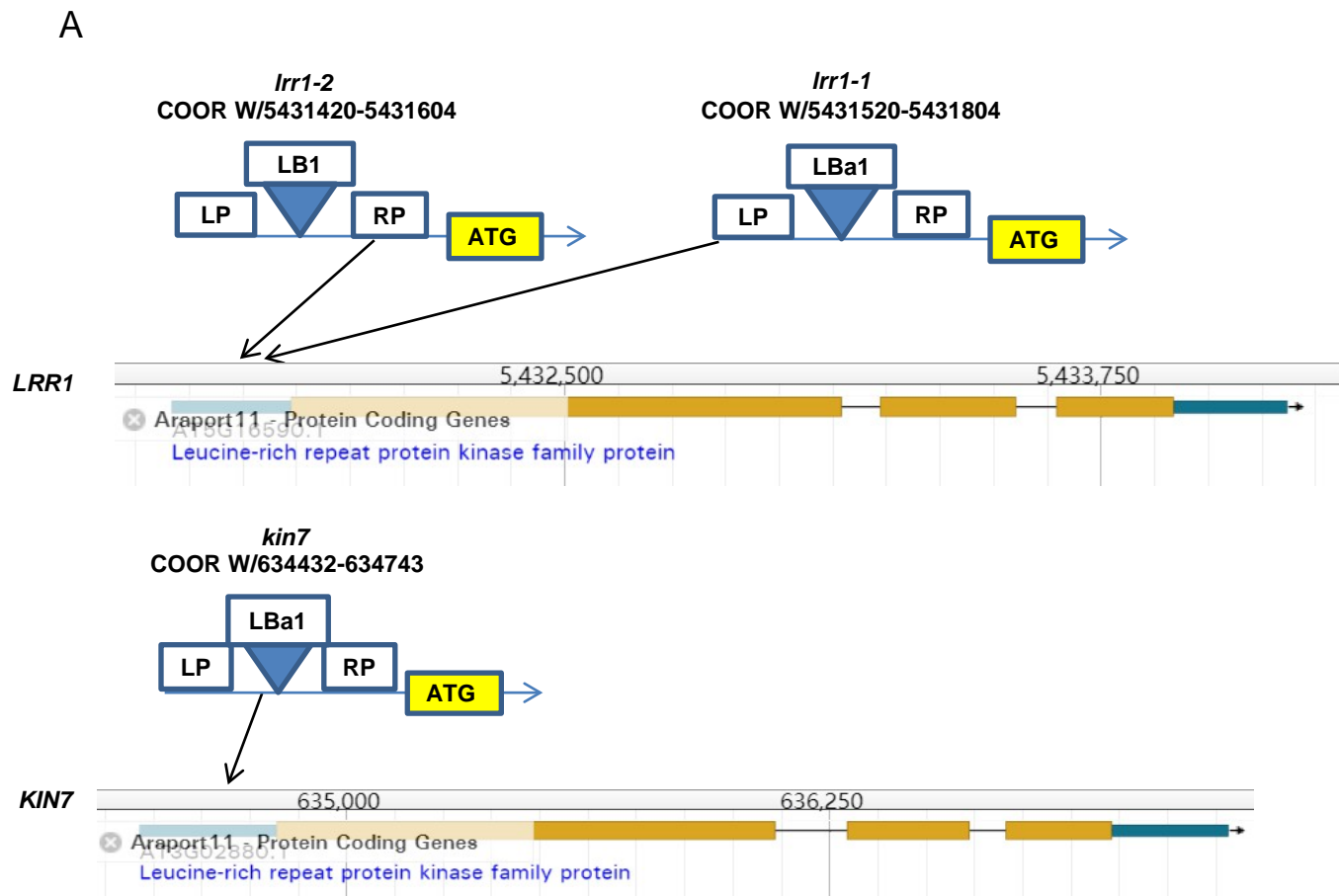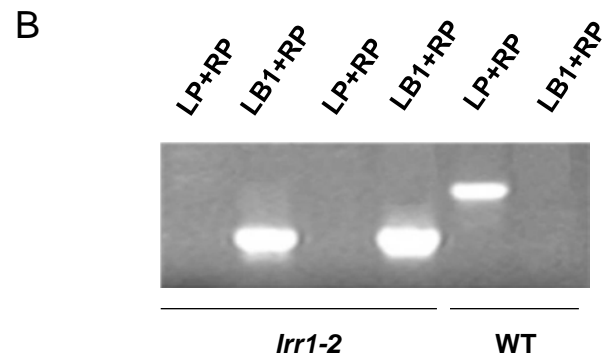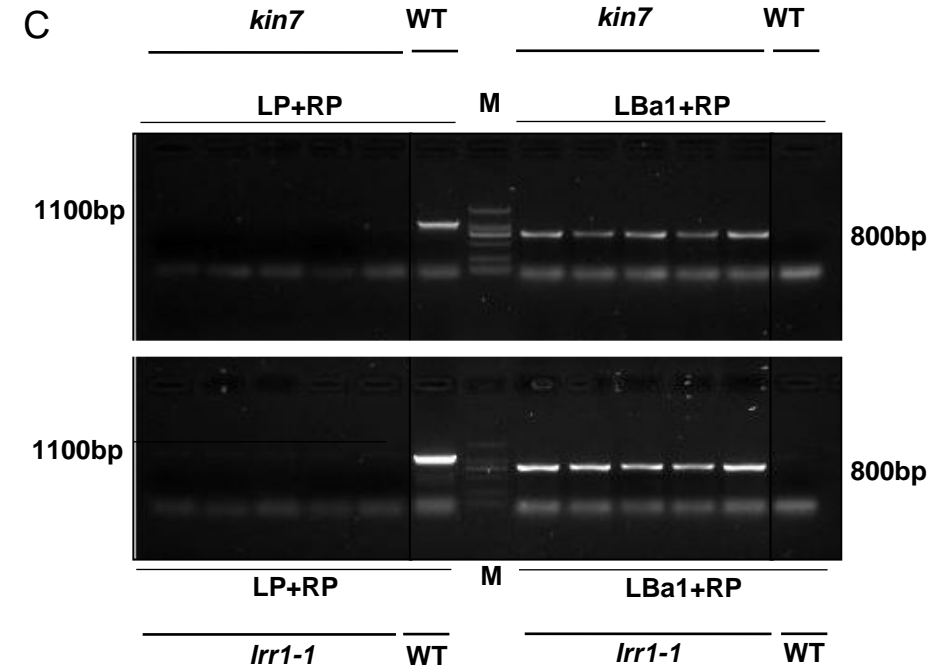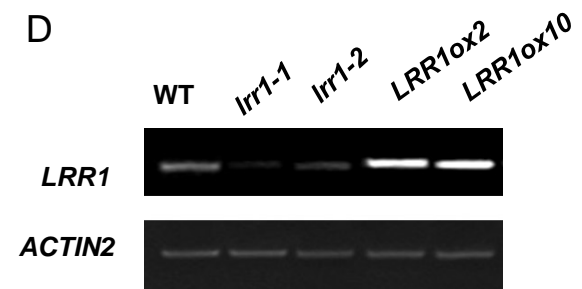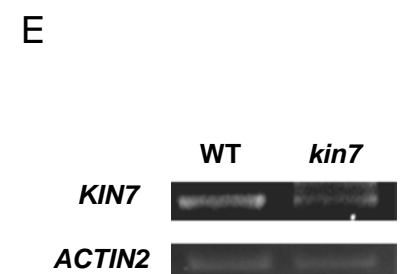

Supplement: Supplemental Information 10 — (A) T-DNA insertion sites for lrr1-1, lrr1-2, and kin7. T-DNA was inserted near the promoter region 1000 bp upstream of the ATG, respectively. (B) Mutants lrr1-2 using LP+RP/LB1+RP as primers. lrr1-2 (SAIL_412, LP:5′CCATGAAATAAAAGGGGCTTC3′, RP: 5′GACTCGGAGATTGGTTTTTCC3′, LB1:5′TCAAACAGGATTTTCGCCTGCT3′). (C) Double mutant lrr1 kin7 using LP+RP/LBa1+RP as primers. lrr1-1 (Salk_053366, LP: 5′GCTGGGGGTAAAGAATGAGAC3′, RP:5′ATTCTTCGTCTCCTTGGTTCC3′, LBa1:5′ TGGTTCACGTAGTGGGCCATCG3′), kin7 (Salk_001905, LP: 5′GCTGGGGGTAAAGAATGAGAC3′, RP:5′ATTCTTCGTCTCCTTGGTTCC3′, LBa1:5′ TGGTTCACGTAGTGGGCCATCG3′), M: DNA Marker(100-2000bp). (D) RT-PCR results of expression of LRR1 gene in wild-type WT, mutants lrr1-1 and lrr1-2 , as well as Overexpressed material s LRR1ox2 and LRR1ox10. (E) RT-PCR results of expression of KIN7 gene in wild type WT, mutant kin7 . (D&E) RT-PCR assays showed that the expression of T-DNA mutants of LRR1 and KIN7 genes was not completely downregulated. [file peerj-12-18460-s010.pdf]
