# Supplementary material for: LRR1 involved in the abscisic acid signaling pathway to regulate the early growth and development of Arabidopsis thaliana
Source: PeerJ. 2024 Nov 26;12:e18460. doi: 10.7717/peerj.18460 (PMC11606324; doi:10.7717/peerj.18460)
Supplement: Supplemental Information 11 — shows the RT-PCR results of the expression of the LRR1 gene in wild-type WT mutants lrr1-1 Overexpressed materials LRR1ox2 and LRR1ox10 were treated with 50 µM ABA for 12 h. [file peerj-12-18460-s011.pdf]

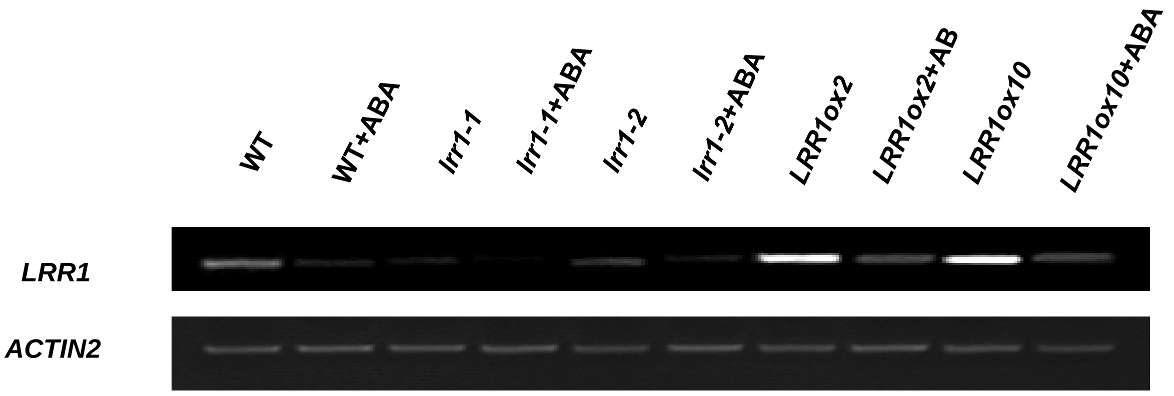
WT
WT+ABA
lrr1-1
lrr1-1+ABA
lrr1-2
lrr1-2+ABA
LRR1ox2
LRR1ox2+AB
LRR1ox10
LRR1ox10+ABA
LRR1
ACTIN2
